# 3D Visual Tracking of an Articulated Robot in Precision Automated Tasks

**DOI:** 10.3390/s17010104

**Published:** 2017-01-07

**Authors:** Hamza Alzarok, Simon Fletcher, Andrew P. Longstaff

**Affiliations:** Centre for Precision Technologies, School of Computing and Engineering, University of Huddersfield, Queensgate, Huddersfield HD1 3DH, UK; s.fletcher@hud.ac.uk (S.F.); a.p.longstaff@hud.ac.uk (A.P.L.)

**Keywords:** Hough transform, visual tracking, eye-to-hand cameras, industrial robotic applications, pick-and-place, target colour selection

## Abstract

The most compelling requirements for visual tracking systems are a high detection accuracy and an adequate processing speed. However, the combination between the two requirements in real world applications is very challenging due to the fact that more accurate tracking tasks often require longer processing times, while quicker responses for the tracking system are more prone to errors, therefore a trade-off between accuracy and speed, and vice versa is required. This paper aims to achieve the two requirements together by implementing an accurate and time efficient tracking system. In this paper, an eye-to-hand visual system that has the ability to automatically track a moving target is introduced. An enhanced Circular Hough Transform (CHT) is employed for estimating the trajectory of a spherical target in three dimensions, the colour feature of the target was carefully selected by using a new colour selection process, the process relies on the use of a colour segmentation method (Delta E) with the CHT algorithm for finding the proper colour of the tracked target, the target was attached to the six degree of freedom (DOF) robot end-effector that performs a pick-and-place task. A cooperation of two Eye-to Hand cameras with their image Averaging filters are used for obtaining clear and steady images. This paper also examines a new technique for generating and controlling the observation search window in order to increase the computational speed of the tracking system, the techniques is named Controllable Region of interest based on Circular Hough Transform (CRCHT). Moreover, a new mathematical formula is introduced for updating the depth information of the vision system during the object tracking process. For more reliable and accurate tracking, a simplex optimization technique was employed for the calculation of the parameters for camera to robotic transformation matrix. The results obtained show the applicability of the proposed approach to track the moving robot with an overall tracking error of 0.25 mm. Also, the effectiveness of CRCHT technique in saving up to 60% of the overall time required for image processing.

## 1. Introduction

In robotics, the use of vision sensors in industrial tasks such as welding, drilling and pick-and-place tasks has become widespread, as the feedback obtained from these sensors make the handling of unknown and dynamic environments possible. Traditionally, industrial robots have the capability to change their position repeatedly with a small positioning error of 0.1 mm, but their absolute accuracy can be several millimetres because of tolerances, elasticities, temperature, etc. [1]. These source of errors can cause a significant offset to the robot end-effector. Therefore, it is important to measure its end-effector position and orientation in Cartesian space [2]. There are two possible solutions for estimating the robot position. The first solution is to use inertial sensors for measuring the position of the articulated robot, for instance by using accelerometers [3]. The other solution is to estimate the robot position globally using external sensors such as laser trackers and intelligent global positioning systems (iGPS) [4]. The advantage of these measuring systems is in their capability in providing sufficient and reliable way to track the robots. However, these systems are more suited for large dynamic tracking measurements rather than small workpiece volumes, in addition to extra hardware requirements that must be installed in order to cover the desired measurement volume and the number of measurement points which raise the overall hardware cost of the tracking systems. Over the last twenty years and among popular sensory systems used for tracking tasks, the vision sensors have emerged as suitable sensors for measuring the position of objects [5] due to their non-contact features and hardware costs. Vision sensors have been widely preferred as tracking systems in different robotic tasks, such as in robotic interception and grasping tasks where they are employed as navigation guidance systems for tracking and intercepting of moving targets [6,7], in robotic welding tasks where are used to perform real time tracking for the weld seams [8,9], and in robotic drilling process for locating reference holes [10,11]. They have the advantage of being cost effective since the cost of image processing has reduced, self-contained systems and also their ability for providing relevant information on the status of robots and their immediate physical environment. The significance of having visual information stands out in applications with unstructured or changing environments [12]. 

In the aforementioned manufacturing tasks, the vision sensor is required to identify and track moving targets, the tracked target can be the object to be manipulated by the robot for example from the conveyor or it can be the robotic gripper (or the end-effector). There are two main performance requirements for applying vision systems in robotic tasks which are: (1) high accuracy of the output results and (2) a fast response of the tracking system. In other words, the accuracy and time efficiency are important, particularly in real world applications. However, achieving the two requirements together is very challenging due to the fact that more accurate tracking tasks are often performed at longer processing time, while quicker responses for the tracking system are more prone to errors, therefore the trade-off between the accuracy for speed, and vice versa is required.

This paper is organised as follows: Section 2 reviews the related work for coping with the accuracy and efficiency issues in the visual tracking task. Section 3 introduces the task challenges and introduces a system that tackles the residual problem. Section 4 describes the concept of the proposed tracking system. Section 5 presents results obtained using the visual tracking system. Section 6 summarises the main achievements of the proposed approach and discusses how the system will be developed for further work.

## 2. Related Work

Different works have been introduced for improving the detection and tracking accuracy of the vision system. For instance, Xu et al. [9] introduced an accurate visual tracking system for robotic welding tasks (within ±0.3 mm in the robotic GMAE), by choosing a suitable optical filter based on the analysis of the spectrum of the welding process and using a calibrated camera. However, they stated that there is a low image capturing rate and low frequency were obtained due to the low hardware cost for their USB camera sensor. Mei et al. [11] proposed an in-process robot based calibration technique using a 2D vision system. The proposed technique was designed to improve the positioning accuracy of a mobile robot drilling system for a flight control surface assembly, the results showed the affectivity of their proposed calibration technique in improving the tracking accuracy of the drilled holes from 4 mm to 0.6 mm. Zhan et al. [13] used a calibrated monocular vision system mounted on the robot arm for providing a small field of view (approximately 18°) in order to obtain good measurement accuracy of 0.15 mm, despite the capability of their detection algorithm (saliency-snake) to detect the reference holes on different noisy captured images, no mention was made on the detection speed of the algorithm during the online tracking process.

Detecting the object to be manipulated in every captured frame is one of concerns affecting the time-efficiency of the vision sensor, especially when the object is slowly moving on the conveyor, therefore, Guo et al. [14] used an embedded image card with a Field Programmable Gate Array (FPGA) for accelerating the processing speed and for enhancing the real-time performance. However, processing of multiple consecutive frames is not the only challenge for performing an efficient tracking task. The computational cost of the tracking algorithm is another source of deficiencies of the tracking system. Feature extraction algorithms such as Hough transforms have been widely used for identifying targets based on point and line features [15,16,17]. The problem with the use of such algorithms is in their significant computational cost [18], which prevents their execution in real-time by a single processor via software [19]. According to Chang et al. [20], the edge detection phase itself takes 80% of the computational time during the estimation of the 3D pose of the target, therefore, Kragic and Christensen [21] enhanced the computational speed of their tracking system by initialising a template matching algorithm in the region where the area of interest was found in the previous frame instead of applying the algorithm on the full image. However, no mention was made in the presented work of whether the accuracy of their pan-tilt camera has any effect on the tracking performance, although the camera had low resolution (around 0.08 Megapixels) and its view to the measurement volume was from a distance of about 2 m.

Some researchers have enhanced the frame rate speed of their visual tracking systems by reducing the camera resolution, such as Gupta et al. [22] who noted that when the camera was used at high resolution (3 Megapixels), a significant reduction in the frame rate occurred (1–2 fps) which caused a low accuracy of the localisation and the tracking system. To cope with the processing speed problem the image resolution was reduced from 3 to 0.5 Megapixels, and in order to enhance the tracking accuracy, a Kalman filter was used. Although the results showed the effectiveness of the proposed method for tracking and detecting the pose of the robot in indoor environments, the incorrect detection of markers in the camera image due to environmental changes is still a remaining problem. Another way for reducing the computational cost of the visual tracking system was introduced by Gengenbach et al. [23] who neglected the effect of lens distortions on the sensor data, and added a fast velocity estimation algorithm based on optical flow vectors. The work aimed to achieve a positioning accuracy of about 1 mm, however, there was no indication about the actual accuracy achieved. The use of a small sized observation window instead of processing full resolution images for reducing the amount of processing data during the tracking process is another possibility, Lund et al. [24] and Shen et al. [25] used a small observation window which is placed around the estimated position of the robot. However, in their work they cited the additional problem of the target being larger than the size of the search window, which can lead to incorrect results. 

## 3. Task Challenges

From the reviewed works, it is evident that recent research has focused on one or the other of the two main performance considerations. One ignores the issue of the high accuracy to achieve the adequate speed required by the given task, whereas the other trades off the time-efficiency for accuracy to execute a precise tracking process. However, in many precision manufacturing tasks such as pick-and place, welding and drilling tasks, it is important for the vision sensor to deliver accurate output results within a necessary response time. However, achieving both requirements with the vision sensors is not an easy task due to the fact that performing processing operations on consecutive high resolution camera frames is going to be computationally expensive [26]. 

This paper proposes a system that tackles the accuracy-efficiency trade-off problem by introducing and validating an efficient, accurate and robust visual system which ensures achieving a sub-millimetre tracking accuracy and a faster detection process. The proposed system is applied in an unstructured, industrially equivalent environment after taking into consideration different factors that often prevent obtaining such accurate and time efficient performance. In addition to the two performance requirements it is essential that any solution be cost effective. 

A feature extraction algorithm named Circular Hough Transform (CHT) is used to identify a spherical target (a ping-pong ball in this case) of known size and sphericity, the effect of which is discussed in Section 4.1.3. The target is mounted on the robot end-effector in order to obtain its relative position. The reason behind using a simple and known sized object is for the robustness of the segmentation process for the target object’s motion from a background in unprepared industrial environments [27,28] and also to avoid using complex shapes which require robust methods of detecting a target in dynamic environments [29].

In order to increase the computational speed of the tracking system, the proposed visual tracking system is implemented with a new technique for generating dynamic search windows. The technique is named Controllable Region of interest based on Circular Hough Transform (CRCHT). The advantage of this technique is in expanding the use of Circular Hough Transform (CHT) for generating small search windows instead of using full resolution images, in addition to their typical use for detecting edge features. Moreover, the size and location of the generated window by the proposed CRCHT technique are automatically controlled and updated based on the variations in the position and dimensions of the spherical target. The proposed CRCHT technique also differs from previous reviewed window generation methods is in that the tracked target will always be centred in the image without being larger than the size of the generated window.

In order to meet the requirement of a high detection accuracy, an averaging filter was used to filter the raw data obtained from the vision sensor in order to reduce the sensor noise. The parameters of the filter were carefully selected based on the utilisation of the extrinsic camera calibration. The colour of the target object was selected based on a new colour selection process. Moreover, for reliable tracking information, a simplex optimisation technique based on Nelder–Mead method [30] was used for finding the parameters of the camera-to-robot transformation matrix. In addition to aforementioned contributions, a mathematical model is presented for the calculation of the scaling factor which is required for obtaining metric information of the tracked end-effector. The idea of the presented formula is based on using the two cameras for updating the depth information of the tracking system and thus updating the scaling or calibration factors in 3D space.

## 4. Automated Tracking for the Robot End-Effector Position Using Enhanced Hough Transform and Multi Eye to Hand Camera System

The proposed tracking system combines two modules: object tracking module and the position estimation module. The object tracking module consists of Circular Hough Transform (CHT) which was using the Phase Coding method [31], the key idea of the method is in the use of complex values in the accumulator array with the information of the radius encoded in the phase of the array entries. The votes cast by the edge pixels contain information of the circle radius associated with the possible centre locations.

In this work, the CHT algorithm is used for extracting a particular circular feature of the object (a ping-pong ball) obtaining its position along with its motion in pixel coordinates. This information is then passed to the position estimation module in order to calculate the position of the object in world coordinates. The object used is mounted on the end-effector of a six DOF robot (RV-1A, Mitsubishi, Tokyo, Japan) and thus the position of the arm in 3D space can be measured with respect to the target. The details of the two modules are explained in the next sections.

### 4.1. Object Tracking Module

**Figure 1:**
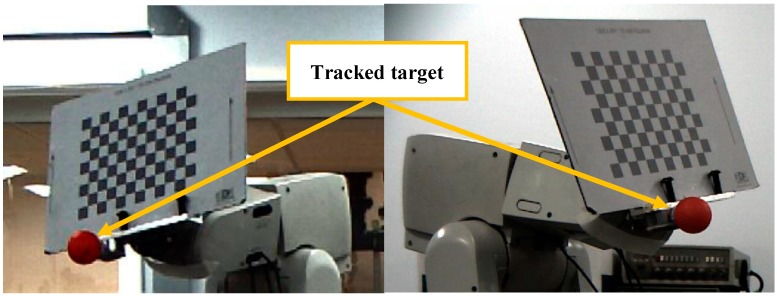
Front view (**left**), side view (**right**).

In this section, the proposed feature extraction algorithm will be described. An enhanced Circular Hough transform algorithm was used to perform the tracking process in two phases: the initialization and tracking phase. During the initialization phase, the algorithm extracts geometric features of the object from the captured images, the two extracted features are the centre and the radius of a ping-pong ball attached to the robot end-effector (see Section 4.1.3 on the influence of the ball’s sphericity). In order to detect the 3D position of the object, the proposed algorithm extracts the location of the ball from one of the two cameras in the XZ plane, while the other camera is used to obtain the location in the YZ plane (see Figure 1). 

During the tracking phase of the process there is a probability that false circles will appear in the captured images due to the nature of the algorithm (phase coding method) used as an edge detector which detects the objects based on their elliptic shape and brightness compared to the background. The brightness of the object to be tracked depends on its colour, therefore in order to reduce the probability of failure in the object detection, the colour of the object should be carefully selected in order to be easily distinguished from the background and reliably detected and thus tracked by the algorithm. 

#### 4.1.1. Colour Selection

This part of the experimental work aims to find the appropriate colour for the tracked object, which ideally should meet two requirements: (1) the colour should be easily recognized and distinguished from the scene; and (2) the colour should be robust to the variations of the light illumination. The latter requirement is challenging for our application due to the fact that the proposed visual tracking algorithm will be applied in working environments.

A 3D laser scanner (namely a Focus 3D X30, FARO, Lake Mary, FL, USA) was used for full scanning of the laboratory environment by taking hundreds of pictures. The importance of this part of the experiment is to find a strong colour that can be recognised without any interference with other colours or lighting and background variation. The lab environment was prepared with different coloured objects such as machines, tables, tissues, computers, coloured papers and etc. The algorithm was used for performing simple colour detection via Delta E colour difference. The algorithm converts images from Red, Green, Blue (RGB) colour space to the LAB colour model, then the difference between colours in LAB space was calculated for every pixel in the image between the colour of the pixel and the average LAB colour for the region specified by the user, so the relative perceptual differences between any two colours in L*a*b* can be calculated by dealing each colour as a point in a 3D space (with three components: L*, a*, b*) and then calculating the Euclidean distance between them. This Euclidean distance in L*a*b* space is ΔE (often called “Delta E”) [32]:

(1) $$\Delta E=\sqrt{{\left(L_{2}^{*}-L_{1}^{*}\right)}^{2}+{\left(a_{2}^{*}-a_{1}^{*}\right)}^{2}+{\left(b_{2}^{*}-b_{1}^{*}\right)}^{2}}$$

**Figure 2:**
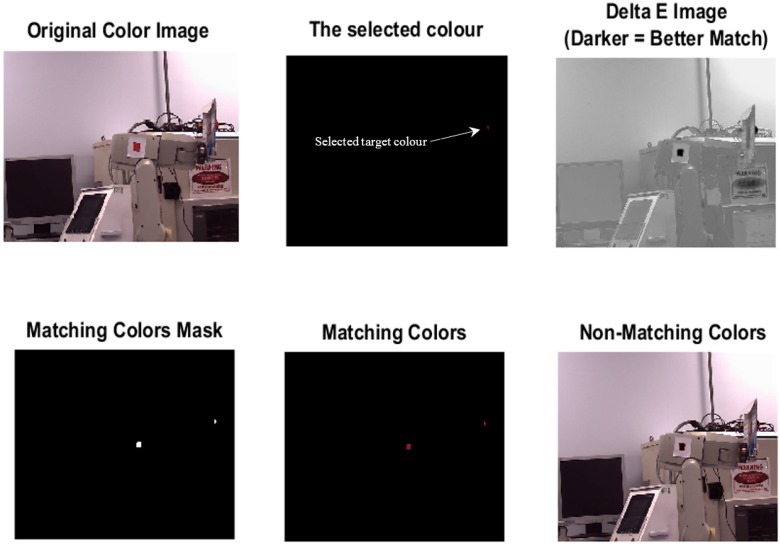
Detection of the red coloured objects.

The best match for the colour of the specified region is represented by the darker colour in Delta E image as shown in Figure 2. Table 1 which shows a list of colours that the algorithm succeeded or failed to recognise them from the background. 

It can be seen from Table 1 that some colours such as red can be clearly recognized among other existing colours in the scene. However, colours such as white and grey cannot be clearly detected because of the interference between the two colours and the lab light. The intensity or the force of the colours also should be taken into account, since the intensity of object’s colour changes according to the location of the object from the camera. Objects with red colour as an example of how the colour can be successfully recognised at different locations in the scene. As a result of this experiment, the red colour was chosen as the colour of our tracked target. 

**Figure 3:**
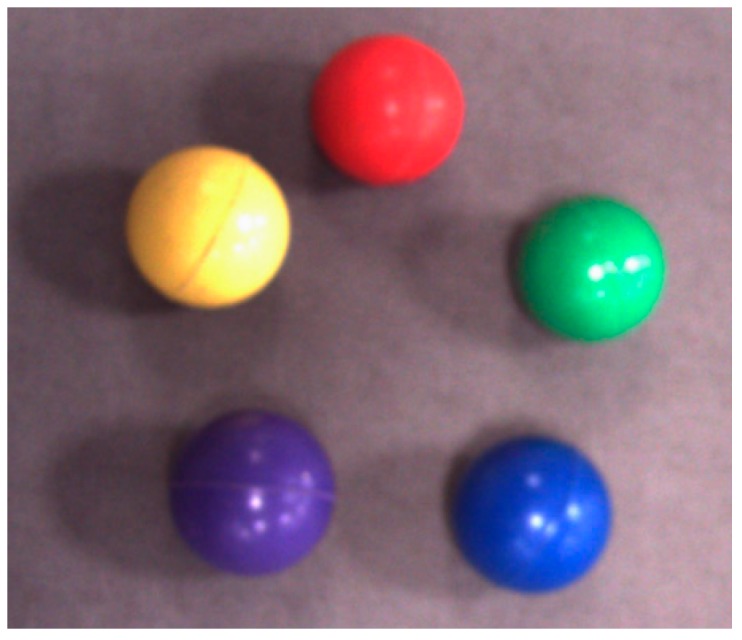
Different coloured objects.

In order to ensure that the selected colour for the object is suitable for the proposed tracking application, different coloured balls (see Figure 3) have been positioned at different eight locations in the lab, and the cooperation of a fixed camera system and the CHT algorithm was used to track the object in a video mode. The experiment has been repeated 20 times under different eight lighting conditions and object backgrounds (i.e., each coloured ball was tested 160 times). Also, at different distances for the objects from the camera’s location (the smallest distance between the camera and the tracked object was around 50 cm and the farthest was approximately 3 m). 

Table 2 shows that the dark colours (blue, purple, and red) possess higher success rate compared to bright colours, and they are robust to the variations in the scene, therefore, the red colour was selected as the colour of the tracked object due to two reasons: (1) the objects having this colour can be easily detected by using CHT with sufficient success rate in tracking tasks; (2) the objects with the similar colour can be recognised and matched in the scene via the Delta E colour difference. Moreover, it is worth mentioning that despite that the blue and purple targets possess the highest success rate, they were not chosen as tracked targets due to that the available balls in these colours had a smaller size and higher roundness error compared to the red one. 

#### 4.1.2. Noise Reduction

The quality of the tracking phase basically depends on the quality of the captured images, so if poor images are obtained, poor object information will be the result. An averaging filter was chosen for reducing random noise without blurring edges. The proper averaging filter was selected based on the utilisation of the extrinsic camera calibration information. The calibration technique introduced by Zhang [33] was used for the camera calibration, and the checkerboard board (as shown in Figure 1) was attached to the end-effector of the robot which is programmed to move and stop at different designated points, three image averaging cases were tested (8, 16 and 32), and a laser tracker was used as a reference for evaluating the performance of the three filters. The filter using an average of 16 images was selected because it provided the highest metric positioning accuracy compared with the other filters, the overall detection accuracy of the vision sensor was improved by 26% when the averaging of 16 images was applied instead of four averaged images. The experiment also showed that using a higher number of averaged images does not always guarantee better detection performance [34].

#### 4.1.3. Roundness Measurement

In addition to the effect of noise on the detection accuracy of the algorithm, if the tracked target used has any deviation from a circular shape (roundness error), the algorithm will fail in finding all border pixels leading to incorrect computation for the centre of mass. In order to determine the influence of such errors, a coordinate measuring machine system (namely a Ziess Prismo Access CMM, Carl Zeiss, Oberkochen, Germany) with an accuracy of 1 µm (for the working volume used in the evaluation) was used to measure the true roundness errors for the ball. A series of roundness measurements were chosen rather than sphericity due to the available characteristics calculated by Calypso metrology software. According to recommendations introduced and proved by Gapinski and Rucki [35] for the roundness measurement with CMM, they stated that in the case of using a minimal number of probing points which is 4 for the circle, too large dispersion in the measurement results will occur both for the centre of the circle and for the form deviation, therefore a large number of probing points (around 6000 points) are collected from the profile of the round object in the scanning measurement. An efficient and robust algorithm named Least Squares Circle (LSC) method was employed for evaluating the actual roundness error. Table 3 shows the roundness measurement of two ping-pong balls obtained by CMM, the ball with the smallest roundness error (0.1134 mm) was selected to be the target object in our tracking application. The detailed results on the effect of the roundness error will be presented in Section 5.

### 4.2. Generating a Dynamic Search Window

**Figure 4:**
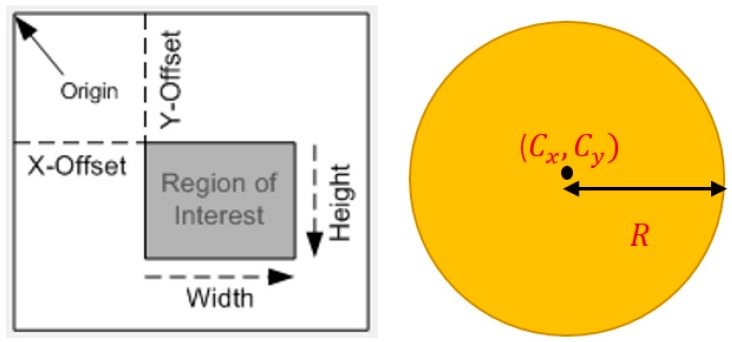
The size and position of the CRCHT window.

The final procedure of the tracking phase is the use of a dynamic search window instead of a full resolution image in order to minimize the processing delay and enhancing the image quality. The positions of the search windows are adjusted in the video frame by the offsets of the area of interest which is calculated from the extracted features for the tracked object, the size and position of the search window are automatically updated along the tracking period based on the variations in the diameter and the centre of the tracked object (see Figure 4), this method expands the use of the CHT algorithm to generating of dynamic observation windows and thus there is no need for applying the window generation method in a separate stage, therefore it is named in this paper as a Controllable Region of interest based on Circular Hough Transform (CRCHT).

**Figure 5:**
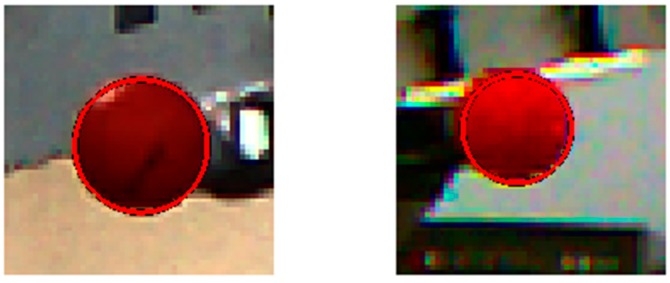
Two CRCHT search windows.

The size of the image can be automatically updated based on the variation in the diameter of the spherical target, and can also be specified by the user by defining the number of rows (Height) and columns (Width). Moreover, the location of the search window is automatically verified based on the change in the centre of the ball (see Figure 5). Therefore, the proposed method generates a dynamic window. 

The following equations are used for the calculation of the position of the generalised observation window:

(2) $$X\_\mathrm{Offset}=C_{x}-2\times R$$

(3) $$Y\_\mathrm{Offset}=C_{y}-2\times R$$

**Figure 6:**
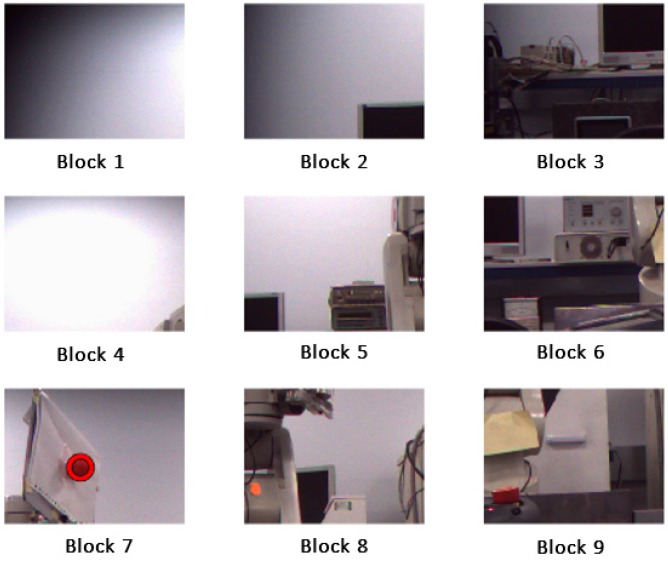
Block processing of the captured image.

**Figure 7:**
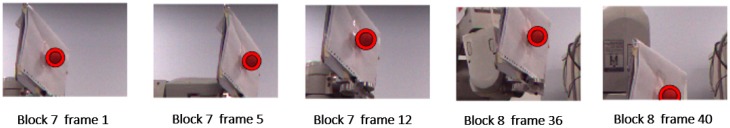
Search windows generated by IIM.

The performance of the CRCHT method will be compared to another method which generates small sized static observation windows named Image Indexing Method (IIM), the concept of the method is based on dividing the image into small blocks (see Figure 6), and process those individual blocks using matrix indexing to get chunk of block data and process them in a sequential manner. Since the IIM generates static windows, therefore, in order to change the location of the generalised window during the tracking process, each individual block (window) is made as a function of time, so the windows or blocks can be varied at specific times which are set by the user, therefore, priori knowledge of the time required for the target to move from one block to another is important. Figure 7 shows how the location of the search window was changed twice based on the change in the location of the robot. 

The CRCHT method was preferred in our 3D target tracking task due to its ease of application, and also for its capability to generate dynamic windows which their size and location are automatically updated along the tracking process without any need for managing the time required to change between individual blocks.

### 4.3. Position Estimation Module

**Figure 8:**
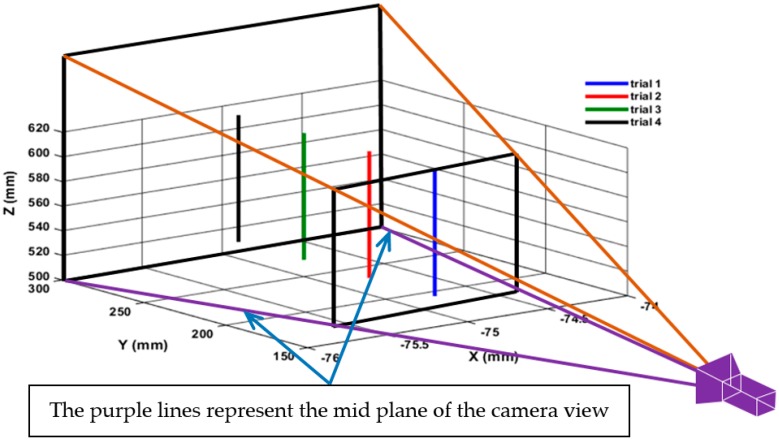
Nominal movements of the target.

In this section, we describe how to estimate the position of the robot end-effector. Since the position of the tracked object can be measured in pixel coordinates, therefore, the calibration factor (or scaling factor) that defines the relationship between the image coordinates (pixels) and world coordinates (mm) has to be known (due to the constantly changing depth of field). The error of the changing depth of field on tracking accuracy was first investigated by moving the target via the robot a distance of 100 mm in Z axis, and measuring the travelling distance by both the laser tracker and the vision system which only provides information in XZ plane. The same travelling distance for the target was repeated 4 times at different depth information (see Figure 8) which is obtained by changing the location of the target from the centre view of the camera (i.e., each trial starts at a different y coordinate as shown in Figure 8), since the change in the Y coordinates of the target cannot be detected from the 2D view of the camera, its influence on the readings of the tracking system can be notified in Table 4 which shows how the change in the depth information of the target caused a positioning error of around 10 pixels which is approximately equivalent to 10 mm when the robot travels from its initial location by 120 mm. 

### 4.4. Camera to Robotic Arm Calibration

**Figure 9:**
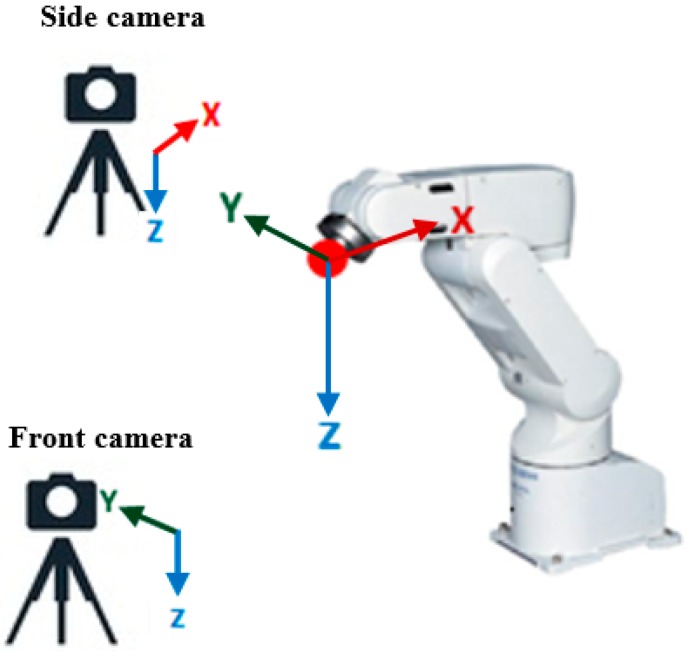
Camera setups in the experiment.

**Figure 10:**
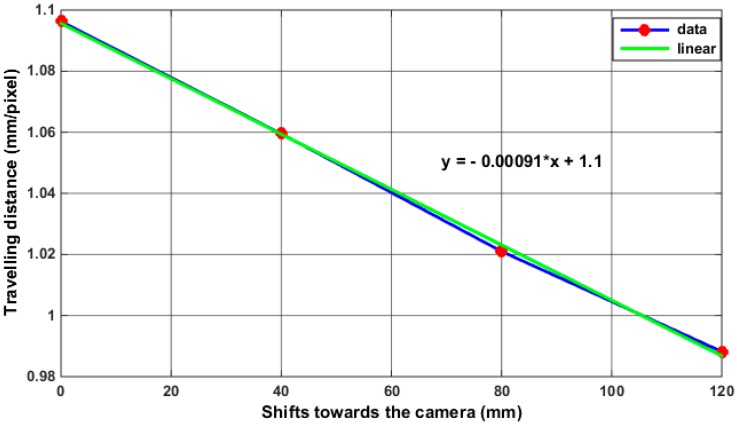
Measurement of the calibration factor in X axis.

We take the tracking problem a stage further by merging the 2D observations from each camera view to form global 3D world coordinates for the object locations. Once the 2D observations have been merged it is then possible to track each target object in explicit 3D coordinates. The use of a single vision sensor can only provide 2D information about the tracked object, therefore in order to extract 3D visual information, a multi vision system is required. However, obtaining this information in real world units requires calculating of the calibration factors in the X, Y and Z axes, and in the case of the tracked object which is changing its location whether moving close or far from the camera centre view, the calibration factor will then be constantly varying., This means that the calibration factor values should be updated based on the motion of the target during the tracking process. In our proposed tracking system, a mathematical formula is introduced for updating the calibration factors based on utilizing the ability of each camera in updating the depth information for the other, since the side camera only can provide information in XZ axis (see Figure 9), the variation in the position of the target in Y axis cannot be known through the side camera itself, and this change will lead to variation in the scale factor for the other two axes. However, the changes in Y axis can be detected and measured by the second camera (frontal camera), therefore, the information of Y axis extracted from the frontal camera will be used to update the value of the calibration factors in XZ axis. A similar procedure will be performed for updating the calibration factor in YZ axis for the frontal camera, the information of the X axis obtained by the side camera will be used to update the calibration factors in YZ axis for the frontal camera. The mathematical formulas used for updating the calibration factors in 3D space can be represented by the simple linear equation (*y* = m × x + c), with gradient or slope m which represents the value of the calibration factor and intercept c. Figure 10 shows an example calculation of the calibration factor in the X axis direction from the relationship between the changing depth of field and the travelling distance of the target (mm/pixel). 

The figure shows that the geometric relationship between the travelling distance (mm/pixel) and the changing depth (mm) is linear. We estimate the error in the fit at these points is just 10 microns per 1 m, we therefore deemed four points to be sufficient. The following three equations were added to the tracking algorithm and used during the tracking process for obtaining the 3D metric information of the tracked object:

(4) $$X\left(i\right)=x\left(i\right)\times \left(m_{x}\times \left(y\left(i\right)-y\left(i-1\right)\right)+c_{x}\;[\text{Side camera}\right]$$

(5) $$Y\left(i\right)=y\left(i\right)\times \left(m_{y}\times \left(x\left(i\right)-x\left(i-1\right)\right)+c_{y}\right)\;[\text{Front camera}]$$

(6) $$Z\left(i\right)=z\left(i\right)\times \left(m_{z}\times \left(x\left(i\right)-x\left(i-1\right)\right)+c_{z}\right)\;[\text{Front camera}]$$

where (X,Y,Z) represent the coordinates of the object in world units, (x,y,z) refers to the coordinates of the object in pixels, i is the number of the captured frame, and ($m_{x},m_{y},m_{z})$ are the calibration factors in XYZ axis. 

In this work we have used an established homogeneous transformation that we have separated in order to highlight the parameters that will be optimised subsequently. In order to find the coordinates of the robot end-effector from the known coordinates of the tracked target, a homogeneous transformation defined by a 4 × 4 matrix was performed by the applying rotation given by R(α, β, γ), then followed by a translation given by $x_{t},y_{t},z_{t}$. The result is:

(7) $$T=(\begin{array}{c} \cos\alpha \;\cos\beta \\ \sin\alpha \;\cos\beta \\ -\sin\beta \\ 0 \end{array}\;\begin{array}{c} \cos\alpha \sin\beta \sin\gamma -\sin\alpha \cos\gamma \\ \sin\alpha \sin\beta \sin\gamma +\cos\alpha \cos\gamma \\ \cos\beta \sin\gamma \\ 0 \end{array}\begin{array}{c} \cos\alpha \sin\beta \cos\gamma +\sin\alpha \sin\gamma \\ \sin\alpha \sin\beta \cos\gamma -\cos\alpha \sin\gamma \\ \cos\beta \cos\gamma \\ 0 \end{array}\begin{array}{c} x_{t}\\ y_{t}\\ z_{t}\\ 1 \end{array})$$

According to Euler 1, the homogeneous transformation matrix can be separated into six parameters $x_{t},y_{t},z_{t},\alpha ,\beta ,\gamma$. It is worth mentioning that the calculation for those elements was automatically obtained by using a simplex optimisation technique which is based on the Nelder–Mead method [30]. After applying the transformation matrix, the results obtained will represent the positions of the end-effector in 3D space, the next step is to measure the positioning error which is the difference between readings obtained from the camera and those measured by the laser tracker. A laser tracker (namely a Faro ION) was used for measuring the actual position of the robot end-effector, it has a distance measurement accuracy (interferometer) of 4 μm + 0.8 μm/m, therefore, one retro reflector (SMR 0.5) was mounted near to the robot end-effector.

In order to obtain the end-effector positions from readings of the laser tracker, the transformation matrix (3 × 1) is necessary to be calculated. The parameters of this matrix represent the translations in 3 dimensions which can be denoted as $x_{t},y_{t}{\;\mathrm{and}\;z}_{t}$.

**Figure 11:**
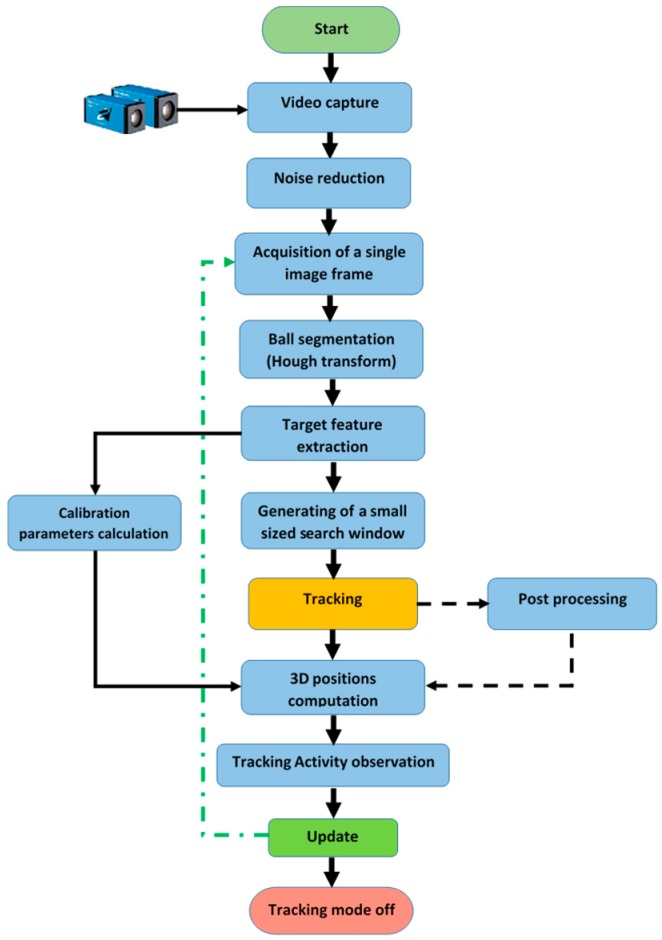
Detailed diagram of the visual tracking system.

Figure 11 shows the detailed diagram of the proposed tracking system, it can be seen that once the features of the tracked object are extracted (i.e., the centre of the ball) from the image, two tasks for the algorithm are performed at the same time, which are generating the two small search windows from the two cameras, and obtaining the position of the robot-end effector in world units by calculating the calibration factors and the transformation matrix.

## 5. Experimental Results and Discussion

**Figure 12:**
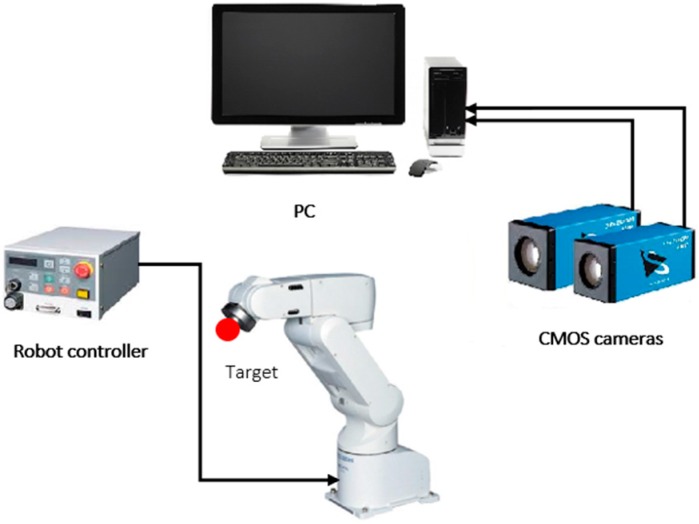
Vision tracking system architecture.

Before describing the results of the experiment, we must describe the architecture of the tracking system (see Figure 12). The system consists of a six DOF robot (Mitsubishi RV-1A, Tokyo, Japan) with a red coloured ball attached to its gripper, a standard PC, and two calibrated CMOS cameras (namely DFK Z12GP031 and DFK Z30GP031) from the Imaging Source (Bremen, Germany) are used for observing and estimating the object motion. The reason behind using colour cameras rather than monochrome sensors is in the capability in using colour filters to control scene contrast, and to keep the other choice applicable which is to convert colour into monochrome. With colour capture, any arbitrary colour filter can be applied in post-production to customize the monochrome conversion, whereas with monochrome capture, the effects of a lens-mounted colour filter are irreversible. The GigE colour cameras are fixed at around 2 m from the object plane and both are connected via Ethernet to the PC, the tracking system was implemented on a Windows environment running on an AMD FX™-9370 Eight-Core Processor 4.4 GHz with 16 GB of system memory. The reason behind the selection of these cameras is due to their distinctive features compared to other manufactured cameras which make them suited for the tracking applications, the main features of the selected cameras can be listed as follow:

- The cameras have an integrated motorized zoom lens, iris and focus that allow for the user to capture objects of differing sizes or when multiple frames at differing magnifications are required.
- The cameras are implemented with CMOS sensors that are highly sensitive sensors making them uniquely suitable for colour based tasks such as component inspection.
- The cameras are supported with a number of powerful software tools such as IC capture and IC measure, the IC capture provides the ability to control the functions of the camera such as auto white balance, noise reduction, and contrast, saturation, and exposure times. The IC measure provides tools for on-screen calibration with an ocular microscope, and provides tools for measuring different shaped objects such as polygons, circles and angles, the measurement tools are designed for both macroscopic and microscopic applications. Also, it offers tools for the optical distortion correction which can corrects barrel, pincushion, and vigneting distortions.

For highly accurate reference measurements, a laser tracker (Faro ION, Lake Mary, FL, USA) was used for evaluating the positioning accuracy of the proposed system. In a tracking system such as the one proposed in this paper, there are various factors that can affect the accuracy of the object tracking, with the main ones being: (1) shape error such as roundness error for the spherical target object; (2) calibration error resulted from following improper procedures for camera calibration; and (3) image process error resulted from the quality of the captured images, the accuracy of the tracking algorithm, and the time delay between capturing and processing the images. Therefore, the effect of these source of errors on the tracking accuracy was taken into our consideration.

(1) Shape or Roundness error**:** the goal here was to measure the roundness error for the target object, as mentioned earlier in Section 4.1.3, a Ziess Prismo Access CMM was used for measuring the roundness errors object by collecting a large number of points from the profile of the object (ping-pong ball), the selected ball for the tracking task (see Table 3) has a roundness error of 0.1134 mm, this value was considered acceptable due to that ball was not particularly made for industrial purposes.

**Figure 13:**
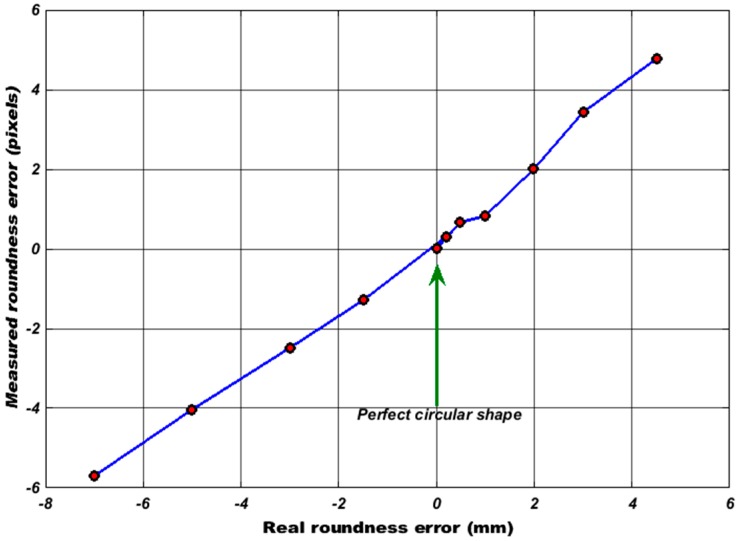
Roundness error measurements.

**Figure 14:**
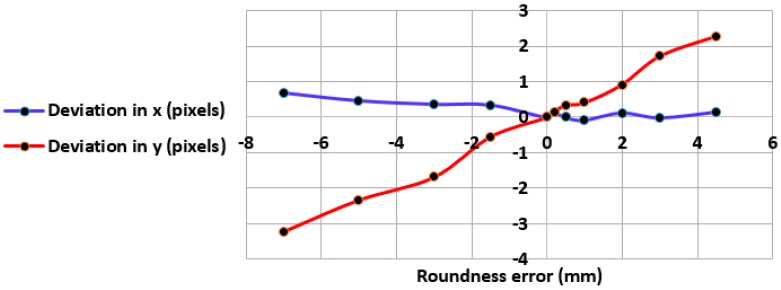
Roundness error versus the estimated measurement deviation.

In order to see the effect of the roundness errors on the measurement accuracy for the tracked target, CHT was applied on different balls, the balls have different values of roundness error, all balls were centred at the same location, and the deviations in their centre readings were calculated from the difference between their measured centres and the centre of the reference ball. It can be seen from Figure 13 that there is a proportional relationship between the actual roundness error measured by CMM and the predicted one by CHT algorithm. Since the selected ball for our tracking application has an actual roundness error of 0.1134 mm, from Figure 14, it can be observed that the target that have a close value of this error will have deviations in its measured position (0.14 pixels in the X axis and 0.15 pixels in the Y axis).

(2) Calibration error: As mentioned earlier, both cameras have been calibrated prior to the experiment based on Zhang’s method, the cameras were fixed at a distance of 160 cm from the checkerboard (shown in Figure 1), since the calibration accuracy for any camera can be evaluated relying on the value of the reprojection error, the mean reprojection error was measured in pixels for the frontal and side cameras is 0.13 and 0.11 respectively. Table 5 shows the results of calibration process for the two cameras.

(3) Image process error: There are three sources for image processing errors which are the poor visual quality of the captured images, the detection inaccuracy of the tracking algorithm, and the time delays between capturing and processing the images, the first source of the error will be neglected as the cameras used are designed for industrial tasks and can provide high resolution images, therefore the detection accuracy of the CHT algorithm and the computational speed of the tracking system will be evaluated.

### 5.1. Evaluation of the Measurement Uncertainties

**Figure 15:**
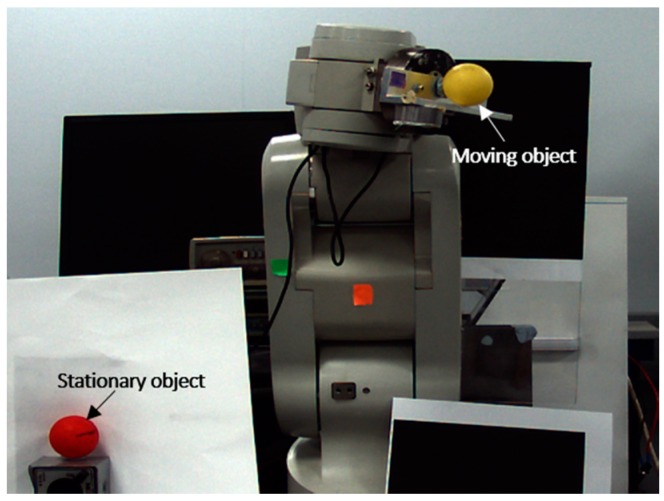
Setup for the still target in the uncertainty test.

The performance of the CHT tracking algorithm was evaluated during the position estimation process for a stationary object, the goal is to examine the detection accuracy of CHT by measuring the uncertainties in pixel coordinates of the target. In order to perform this test, the positions of two cameras and a red coloured ball were fixed along the test. Figure 15 shows the setup of the still target, the ping-pong ball that has an actual roundness error of 0.1134 mm was coloured in red and used as a stationary target in this experimental part, whereas, another ball that has a higher roundness error (0.1994 mm) was mounted on the robot end-effector to clarify the difference between the location of the stationary and moving targets. From Table 6, it can be seen that the measured uncertainties by the two cameras for the object position are quite close in the horizontal axes (0.061 mm and 0.062 mm for the frontal and side cameras). 

Moreover, the correlation percentage of 96% was obtained between the positioning measurements of the object in the Z axis for the two cameras, which indicates to a strong relationship between the measured locations by the two cameras in their common axis. However, it can be seen that the uncertainty values by both cameras in the vertical axis are larger than in the horizontal axis because of the influence of depth variation in this axis is bigger.

### 5.2. Performance Evaluation of the CRCHT Method

**Figure 16:**
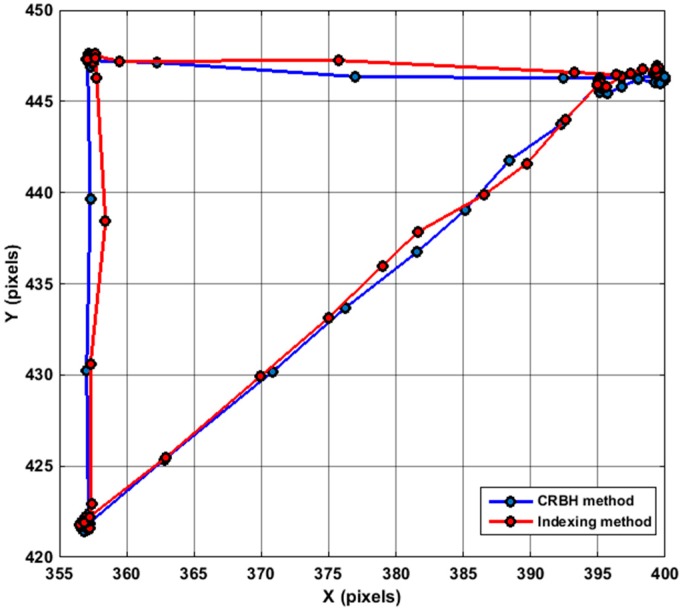
The performance of the two presented techniques in 2D tracking process.

A single camera of 768 × 576 (442 KB) was used to examine the performance of the presented CRCHT method against the performance of the Image Indexing method for generating a small observation window around the estimated position of the tracked object. The performance of the two methods was compared during an online 2D tracking experiment, both use the same camera setup and also with same predefined trajectory path for the robot. Figure 16 shows the robot path for the two techniques in the trajectory tracking of the moving target in 2D space.

Although Table 7 shows that the frame rate performance of the two methods was similar, in automated 3D object tracking, the indexing method costs longer processing time with less detection accuracy for the tracked target compared with CRCHT method, for instance, when IIM method was used for generating a small observation window of (150 × 150 pixels), the processing time for each captured frame was 0.835 s, whereas the processing time required for the same window size with the CRCHT method was 0.4554 s (see Table 8). Moreover, the slowness in the performance of IIM method is referred to the following three reasons: (i) in the case of a robot having a greater working volume, then the number of blocks used will be increased because of using a single small window cannot continuously provide a full view for the object during the tracking process.; (ii) the synchronization between two cameras is vital, the difficulty is in the individual block processing for two images at the same time, and particularly managing the required time to change between individual blocks; (iii) the difficulty to keep the object tracked during the time of changing between the blocks, particularly when the object might not immediately appear in the next block which leads to failure to find it and thus detecting its position. Moreover, since the indexing method has the property of processing each block individually, the 2D translation matrix should be calculated and applied for each block in order to relate the tracking information for the object in these blocks. The data processing will be more difficult in the case of the 3D position of tracked object is required in world coordinates, as such for this case, a 3D transformation matrix should also be applied. Therefore, the CRCHT method is preferred for the 3D visual tracking application. 

The effectiveness of CRCHT in reducing the processing time for the visual system has been examined, Table 8 shows that generating a small search windows can significantly reduce the time required for processing the captured frames, although in our experimental work, there was no special hardware used for the task of image processing. The use of a small search window (46 × 46 pixels) can reduce around 60% of the overall processing time required for a full resolution image (1280 × 720 pixels). 

**Figure 17:**
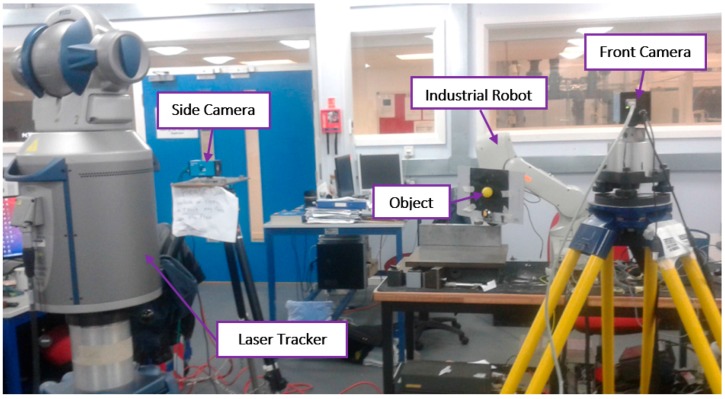
Experiment setup layout.

Summarising the setup layout for the 3D visual tracking experiment (Figure 17), the six DOF robot end-effector was programmed to perform a series of linear motions in a 3D volume, and commanded to stop at predefined picking-up and placing locations while the multi camera system and the laser tracker were used for measuring the position of the robot end-effector along its motion trajectory. An image size of (720 × 576) pixels and a dynamic Region of Interest windowing with a dimension of (65 × 65) pixels have been employed, with a pixel size of 2.2 µm × 2.2 µm. The tracking accuracy of the proposed system was measured from the difference between the reference readings of the laser tracker and those measured by the vision system. It is worth mentioning that accurate positions of the end-effector could be extracted from the robot direct kinematics if a proper geometric calibration for the robot was performed prior to the experiment. However, we cannot rely on data extracted from the robot kinematics since its output results does not represent the actual accuracy of the robot due to the neglecting of the effect of time varying errors such as caused by the variations in the environmental conditions or when the machine warms (thermal errors).

**Figure 18:**
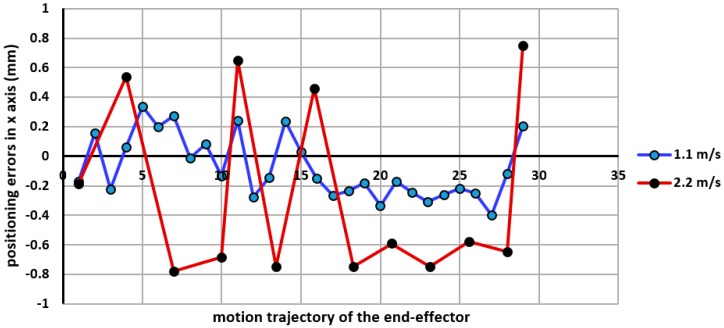
The end-effector travelling speed versus the trajectory accuracy.

The travelling speed of the robot was kept at approximately of 1.1 m/s (i.e., at 50% of the maximum robot speed) in order to give sufficient time for capturing and processing the captured frames. However, in the case when the robot is commanded to travel at higher speeds (for instance at 2.2 m/s), will reveal a much larger tracking error (Figure 18).

**Figure 19:**
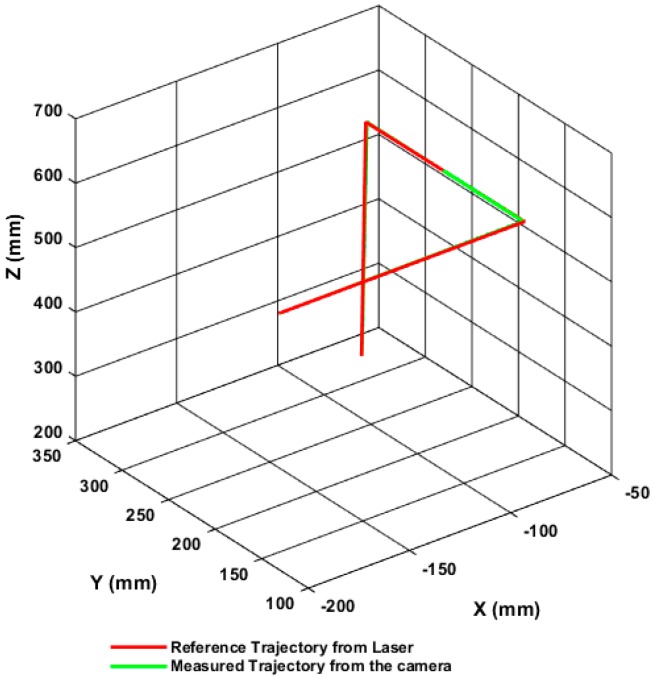
Measured and reference trajectory of the moving robot.

During the performance of a pick-and-place task, the positioning accuracy for the tracked end-effector was examined along its trajectory path. Figure 19 shows the measured and reference trajectory of the robot end-effector four locations which are the pick-up and place locations. The 3D measurements obtained by the proposed tracking system can be seen in Table 9, and the maximum positioning errors along the trajectory path of the robot in 3D space are (0.25 mm, 0.29 mm, and 0.21 mm). 

**Figure 20:**
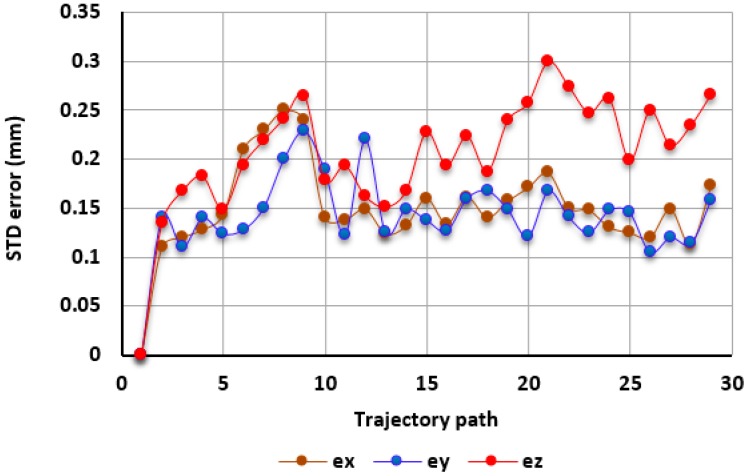
Standard Deviation measured along the robot motion trajectory.

In order to examine the repeatability of the tracking system, the experiment has been repeated 50 times under different light conditions and different daily times (incorporating some environmental temperature variation of up to ± 2 °C), Figure 20 shows the measured standard deviation for the tracked target at each location, the averaged repeatability calculated in XYZ axis is 0.15 mm, 0.14 mm, and 0.21 mm, respectively. However, among the number of performed iterations, there were 20 experiments iterated under the controlled environmental conditions, i.e., the light illuminations and room temperature were kept invariant, and also with no changes made in the robot motion scenario and cameras’ tripods locations, the results showed an improved repeatability value which is 0.085 mm, 0.08 mm, and 0.118 mm. 

**Figure 21:**
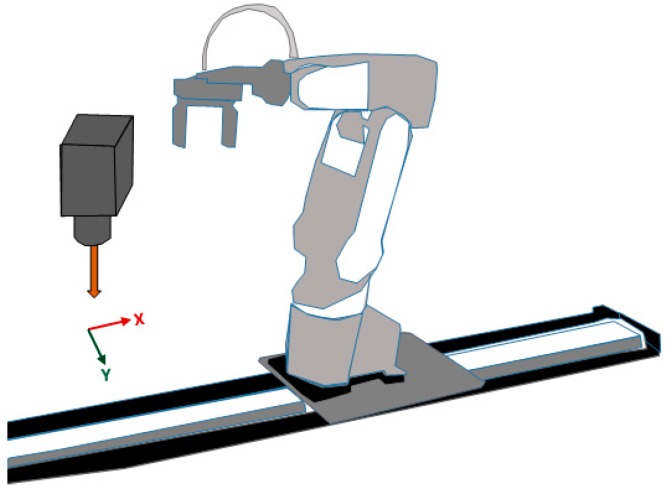
2D visual tracking for a pick-and-place task.

It can be seen from Figure 20 that the maximum positioning error in the XY is smaller than in the Z axis. However, the overall achieved accuracy of 0.25 mm in 3D space was four-times higher than the demands of our pick-and-place task which is within ± 0.3 mm. The plane of interval in XY can be sufficient for tasks where the target is moving on a horizontal plane (such as on the conveyor) and the camera was setup to be perpendicular to the workpiece surface and the distance between camera and the surface is not variant (Figure 21).

## 6. Conclusions

Vision-based tracking research, particularly for precision manufacturing tasks, has typically concentrated either on the requirement of the accuracy or the time-efficiency, therefore, this paper aims to bridge the gap between the two important performance requirements by introducing an accurate and time-efficient tracking system that is also robust in industrial environments. This paper presents and validates an Eye-to-Hand visual system for tracking an industrial manipulator performing a pick-and-place task. The cooperation of Circular Hough transform with an averaging filter is used to obtain the visual information of the tracked target. In order to cope with the computational cost of the image processing, a technique named Controllable Region of interest based on Circular Hough Transform (CRCHT) is introduced for speeding up the tracking process by generating dynamic small search windows instead of using full sized images. The technique is implemented in the proposed tracking system, giving the user the capability of enhancing the time efficiency of the tracking system. Also, in order to enhance the measurement accuracy of the vision sensor, the colour and geometric features of the tracked target are carefully selected, and the cameras are calibrated prior to the experiment. The parameters of the applied averaging filter are also carefully selected based on the utilisation of extrinsic calibration information, and a simplex optimisation technique is used for obtaining an accurate calculation of the transformation matrix. Moreover, this paper also validates a mathematical formula for updating the scaling factors in 3D space which are vital for obtaining 3D metric information for the end-effector of the robot, the presented formula is automatically updated with the variations in the depth information of the vision system. The results showed that the use of the CRCHT technique can save up to 60% of the overall time required for image processing. The experimental results also showed that the proposed Eye-to-Hand system can perform a visual tracking task for the end-effector of an articulated robot during a pick-and-place task with a maximum positioning error of 0.25 mm in the XY plane. For future work, a development exercise for improving the positioning accuracy in the Z axis requires an optimal interaction between three cameras. Therefore, three cameras are further suggested for the 3D tracking task, each camera will be used for providing 1D information of the tracked target. Also, due to the high amount computation to command from the host PC, the CHT algorithm will be implemented in a separate hardware card (a smart FPGA card). Also in order to enhance the processing speed of the proposed system, the search radius range for the CHT will be automatically adjusted and updated for the tracking system via a feedback signal received from the robot controller.

## Figures and Tables

**Table 1:** List of colours and their status.

Colours	The Status	Other Information
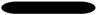	It Can be clearly detected	
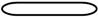	It Cannot be detected	It interferes with grey colour if it is close to the object
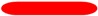	It Can be clearly detected	
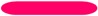	It Can be clearly detected	
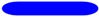	It Can be clearly detected	
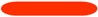	It Can be clearly detected	
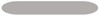	It Cannot be detected	It interferes with white colour
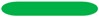	It Can be clearly detected	
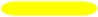	It Can be clearly detected	
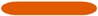	It can be clearly detected	

**Table 2:** Object colour versus the tracking success rate.

Colour	Success Rate
Blue	73.8%
Purple	78.7%
Red	61.25%
Green	35%
Yellow	25%

**Table 3:** Roundness measurement of different balls by CMM.

Objects	Actual (mm)	Tolerance	Actual Diameter	Nominal Diameter	Points	Speed
1	0.1134	0.0	39.7080	39.70	6926	25
2	0.1994	0.0	39.6257	39.70	6929	25

**Table 4:** The influence of the changing depth on the measurement accuracy of the tracking system.

Changing Depth (mm)	Y Coordinates (mm)	Nominal Z-Distance (mm)	Laser Z-Distance (mm)	Camera Z-Distance (Pixels)
0	170	100	100.387	91.2435
40	210	100	100.556	93.5858
80	250	100	100.566	97.0134
120	290	100	100.647	100.8583

**Table 5:** List of estimated calibration errors for the two cameras.

Estimation Errors	Front Camera	Side Camera
Skew error	6.27	5.66
Focal length error	37.4, 37.9	46.32, 45.02
Principal point error	31.73, 21.42	30.1, 20.1
Radial distortion error	0.051, 0.71	0.042, 0.176
Tangential distortion error	0.0030, 0.0042	0.0029, 0.0053
Mean reprojection error	0.1335	0.1147

**Table 6:** Position estimation uncertainty for a still target.

Locations	Frontal Camera		Left Camera	
	Y	Z	X	Z
1	224.0612	607.4006	225.4768	632.1066
2	224.1915	607.5188	225.5007	632.1444
3	224.2305	607.4707	225.5705	632.1138
4	224.1274	607.2804	225.6459	632.4103
STD (pixels)	0.074	0.104	0.076	0.145
STD (mm)	0.061	0.085	0.062	0.119

**Table 7:** Comparison of the processing speed for CRCHT and Indexing method.

Method	Indexing Method			CRCHT Method (56 × 56)
	One Window (384 × 288)	Two Windows (384 × 288)	One Window (59 × 57)	
Elapsed Time (s)	91.4142	91.2582	91.5531	90.9750
Time Per Frame (s)	1.4064	1.3827	1.2895	1.5685
Effective Frame Rate	0.7110	0.7232	0.7755	0.6375

**Table 8:** Window sizes versus the processing speed of the images.

Window Size (Pixels)	(46 × 46)	(66 × 66)	(150 × 150)	(1280 × 720)
Time per frame (s)	0.4418	0.4492	0.4554	0.7084

**Table 9:** 3D measurements of the tracked end-effector at pick and place locations.

NO	ROBOT (Nominal mm)			LASER (Actual mm)			Camera (Measured mm)		
Locations	X	Y	Z	X	Y	Z	X	Y	Z
1	−200	130	600	−200	130	600	−200	130	600
2	−80	130	600	−79.36	129.63	600.79	−79.70	130.18	601.14
3	−80	300	600	−79.38	300.2	599.19	−79.02	299.96	598.97
4	−80	300	250	−83.2	296.39	246.35	−82.87	296.78	246.62
